# Investigating the mechanism of interfacial tension reduction through the combination of low-salinity water and bacteria

**DOI:** 10.1038/s41598-024-62255-0

**Published:** 2024-05-18

**Authors:** Arastoo Abdi, Behnam Ranjbar, Yousef Kazemzadeh, Farzaneh Aram, Masoud Riazi

**Affiliations:** 1 IOR/EOR Research Institute, Enhanced Oil Recovery (EOR) Research Center, Shiraz University, Shiraz, Iran; 2 Department of Petroleum Engineering, Faculty of Petroleum, Gas, and Petrochemical Engineering, Persian Gulf University, Bushehr, Iran; 3 Biotechnology Institute, College of Agriculture, Shiraz University, Shiraz, Iran; 4 School of Mining and Geosciences, Nazarbayev University, Kabanbay Batyr 53, Astana, 010000 Kazakhstan

**Keywords:** Low salinity water, Asphaltene, Intrinsic surfactant, Biosurfactant, IFT, Engineering, Chemical engineering

## Abstract

In the enhanced oil recovery (EOR) process, interfacial tension (IFT) has become a crucial factor because of its impact on the recovery of residual oil. The use of surfactants and biosurfactants can reduce IFT and enhance oil recovery by decreasing it. Asphaltene in crude oil has the structural ability to act as a surface-active material. In microbial-enhanced oil recovery (MEOR), biosurfactant production, even in small amounts, is a significant mechanism that reduces IFT. This study aimed to investigate fluid/fluid interaction by combining low biosurfactant values and low-salinity water using NaCl, MgCl_2_, and CaCl_2_ salts at concentrations of 0, 1000, and 5000 ppm, along with *Geobacillus stearothermophilus*. By evaluating the IFT, this study investigated different percentages of 0, 1, and 5 wt.% of varying asphaltene with aqueous bulk containing low-salinity water and its combination with bacteria. The results indicated G. *Stearothermophilus* led to the formation of biosurfactants, resulting in a reduction in IFT for both acidic and basic asphaltene. Moreover, the interaction between asphaltene and *G. Stearothermophilus* with higher asphaltene percentages showed a decrease in IFT under both acidic and basic conditions. Additionally, the study found that the interaction between acidic asphaltene and *G. stearothermophilus*, in the presence of CaCl_2_, NaCl, and MgCl_2_ salts, resulted in a higher formation of biosurfactants and intrinsic surfactants at the interface of the two phases, in contrast to the interaction involving basic asphaltene. These findings emphasize the dependence of the interactions between asphaltene and *G. Stearothermophilus*, salt, and bacteria on the specific type and concentration of asphaltene.

## Introduction

Smart water is an enhanced oil recovery (EOR) process that modifies the composition of the injected brine to improve the recovery factors. There are two types of smart water: low-salinity water (salinity < 5000 ppm) and high-salinity water. The injection of low-salinity water was a beneficial method approximately 54 years ago^1^. However, unlike sandstones, carbonate reservoir mechanisms present some inexplicable phenomena that hinder the efficacy of low-salinity water^2^. While numerous studies have identified wettability alteration as the primary mechanism, other factors have also been investigated because of the emerging nature of low-salinity processes^1,3^. Therefore, the primary objective of smart water is to alter the wettability from oil-wet to more water-wet. In addition, interfacial tension (IFT), a critical parameter, influences the perception of wettability alteration. By decreasing the IFT, the capillary number increases, resulting in EOR and diminished residual oil^4–6^. IFT is influenced by various parameters, including the presence of asphaltenes, total acid number (TAN), salinity, pressure, and temperature^3,7^. Consequently, the interaction between these parameters is a topic of debate. In addition to low salinity, different concentrations of asphaltenes can contribute to IFT reduction. Lashkarbolooki et al.^8^ evaluated and investigated the impact of asphaltene and resin, along with different salt concentrations, on acidic oil and brine solutions. Their analysis indicated that asphaltenes were more efficient than resins in reducing IFT^8^. In the same study, Lashkarbolooki and Ayatollahi^3^ investigated the simultaneous effect of asphaltenes and salinity, which yielded similar results to those of the previous article^3^. Using IFT measurements, Lashkarbolooki and Ayatollahi^3^ evaluated three crude oils with varying concentrations of asphaltenes and resins. They found that increasing the aromaticity of asphaltenes weakened the decreasing trend of IFT, whereas the opposite was observed for resins^9^. Soleymanzadeh et al.^7^ studied the influence of salinity and asphaltenes on IFT (brine/oil). They observed a direct relationship between the increase in salinity and the corresponding increase in IFT. They also investigated the role of asphaltenes in the presence of other fractions, which reduced IFT under unstable oil conditions. However, under stable conditions, asphaltene particles precipitated, resulting in decreased concentrations at the interface and an increase in IFT^7^.

Different studies report varying results for the IFT between brine and oil, and no consistent trend is observed between salt concentration and IFT values. One of the key factors influencing IFT is the characteristics of the asphaltene and resin fractions^9^. Regarding the significance of asphaltene compared with resin, evidence suggests that asphaltene extracted from acidic crude oil plays a more prominent role in reducing IFT. Therefore, the adsorption of asphaltenes and resins at the interface between brine and oil is of great importance^3,8,10–12^. Given the uncertainties surrounding the influential parameters of IFT, it is necessary to evaluate certain factors, such as asphaltene.

Low-salinity water injection offers the advantage of being compatible with other EOR methods. According to Abdi et al.^13^, the combination of low-salinity water and bacteria for fluid–fluid interactions can yield superior results compared with low-salinity water or microbial EOR (MEOR) alone. MEOR applications using bacterial activities and products like biosurfactants reduce IFT and modify wettability^13–18^. However, Alkan et al.^19^ argued that the IFT reduction from the MEOR is often insignificant. As biotechnology engineering continues to advance, more efficient methods are being employed to enhance the effectiveness of microorganisms and their byproducts, making MEOR more economically viable and widespread^20–26^. Microorganisms offer an environmentally friendly approach to converting heavy oil into lighter compounds by breaking down polar fractions, long aliphatic chains, and aromatic rings, thereby reducing oil viscosity and increasing recovery factors^27^. Biosurfactants contribute significantly to IFT reduction by collecting at the interface of two immiscible fluids, thereby lowering surface tension and IFT^28–30^. MEOR methods have become particularly important in high-salinity environments where other EOR techniques, such as chemical methods, may face challenges. In such cases, the use of halophilic bacteria is recommended^31^. Rabiei et al.^17^ achieved a decrease in IFT from 29 to 3.2 mN/m between oil and brine using biosurfactants from *Enterobacter cloacae* and *Enterobacter hormaechei* in in-situ and ex-situ scenarios of core flooding tests. Najafi-Marghmaleki et al.^30^, could effectively reduce the IFT between the crude oil and the formation brine. This reduction was achieved using biosurfactants derived from *Alcaligenes* faecalis. The IFT was successfully lowered from an initial value of 28.1 mN/m to a final value of 8.4 mN/m. In addition, the wettability of the system was improved, transitioning from an oil-wet state to an intermediate-wet state^30^. The use of microorganisms and their byproducts to increase recovery factors through IFT reduction, wettability alteration, and various other mechanisms can be employed independently^30,32–40^ or in combination with other EOR methods^13^.

Recently, researchers have focused on fluid–fluid interaction as the primary mechanism of low-salinity water^41–53^. Recent studies have highlighted the significant role of fluid–fluid interactions in the effectiveness of low-salinity water^41–53^. Wang and Alvarado^54^ provided evidence that fluid–fluid interaction during the injection of low-salinity water contributes to increased oil recovery. Evaluating IFT is crucial for understanding fluid–fluid interaction and its impact on capillary force. The limited reduction in IFT observed in low-salinity water injection (only a few units) has led to the neglect of IFT evaluation in oil recovery resulting from such injection. However, studies by Wang and Alvarado^55^, Cho et al.^56^ and Ch´avez-Miyauchi et al.^57^ have shown that reducing IFT without altering wettability (making it more water-wet) can enhance oil recovery. A key factor determining the effectiveness of low-salinity water in crude oil is the presence of polar components^1^. Asphaltenes, because of their structure, can accumulate at the interface between water and oil, exhibiting surface-active materials^58–60^. This study aimed to assess the significance of IFT in understanding the mechanism of low-salinity water and its impact on enhanced recovery. Given the importance of asphaltene in fluid–fluid interaction, it can significantly affect the performance of low-salinity water. Therefore, this study examines two types of asphaltene (acidic and basic) with varying percentages. Reducing the IFT is crucial for increasing oil recovery, with the degree of reduction playing a key role in determining its effectiveness. Recent studies have shown the dependency of low-salinity water efficiency on IFT reduction. Consequently, investigating the modification of low-salinity water components to enhance oil recovery by reducing IFT is warranted.

The fluid–fluid interaction in low-salinity water has been extensively discussed in the literature, as mentioned earlier. This interaction is considered to be the primary mechanism underlying low-salinity water. However, further studies are needed to validate the existing findings and provide more detailed insights, as the literature on low-salinity water still contains questions and contradictions. The research conducted by Abdi et al.^13^ explored the potential of combining low-salinity water and bacteria for crude oil applications. Abdi et al.^13^ demonstrated that the concurrent use of low-salinity water and bacteria exhibits a heightened capacity to diminish IFT and may surpass the individual effectiveness of low-salinity water or bacteria in isolation . A recent study by Abdi et al.^61^, they investigated the fluid–fluid interaction by measuring the IFT. They aimed to contribute to the existing knowledge on low-salinity water by elucidating the behavior of IFT through the interaction of ion hydration shells with the polar components of oil. This study examines the combination of low-salinity water and bacteria, presenting a novel aspect of the mechanism proposed by Abdi et al.^61^. This study provides a mechanistic understanding of the behavior of IFT in a combination of low-salinity water and bacteria. To ensure a more accurate interpretation of the investigated interactions, synthetic oils with specific percentages of different asphaltenes (polar components) were used in the study because the described mechanism is influenced by the polar components of the oil. This study explores the importance of the asphaltene structure and percentage in the interaction of low-salinity water containing different salts and salinities, in addition to the presence of biosurfactants produced by bacteria. This investigation introduces a novel approach for enhancing the efficiency of low-salinity water and MEOR, which will be further elaborated upon in subsequent sections. This study evaluated different salts at varying salinities, such as low-salinity water, combined with *Geobacillus stearothermophilus* (strain bio14), and various synthetic oils with different asphaltene percentages and types. Several MEOR studies have focused on reducing IFT and improving recovery using *Bacillus strains*^62–65^. Among these strains, the *Geobacillus* genus is notable for its critical features, such as being gram-positive, rod-shaped, chemo-organotrophic, and aerobic or facultatively anaerobic^13^. Moreover, Geobacillus strains are thermophilic, allowing them to tolerate high-temperature environments like oil fields^66–68^. Zargari et al.^69^ first introduced *G. Stearothermophilus* (strain bio14) for MEOR purposes, demonstrating acceptable tolerance to temperature and salinity. Subsequently, Abdi et al.^13^ used *G. Stearothermophilus* (strain bio14) along with the smart water method to reduce IFT and enhance recovery factors.

## Materials and methods

### Salt and salinity

In this study, three salts, namely NaCl, MgCl_2_, and CaCl_2_, were investigated at concentrations of 1000 and 5000 ppm. These salts were evaluated in two sections: low-salinity water and a combination of low-salinity water and bacteria. NaCl, MgCl_2_∙6H_2_O, and CaCl_2_∙2H_2_O salts from MERCK were used to prepare the aqueous phase containing the salt.

### Oils

**Figure 1:**
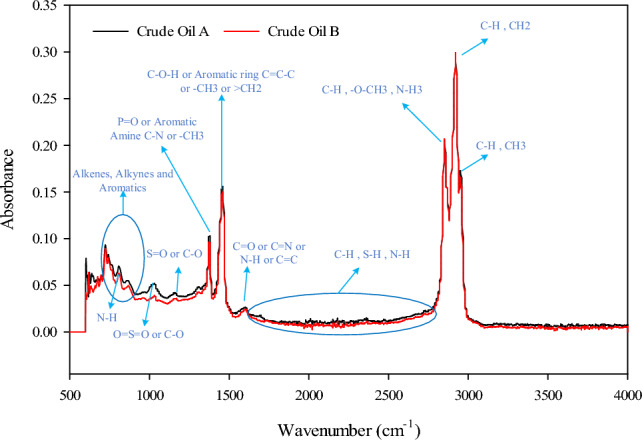
Fourier-transform infrared spectroscopy (FTIR) of crude oils^13^.

**Figure 2:**
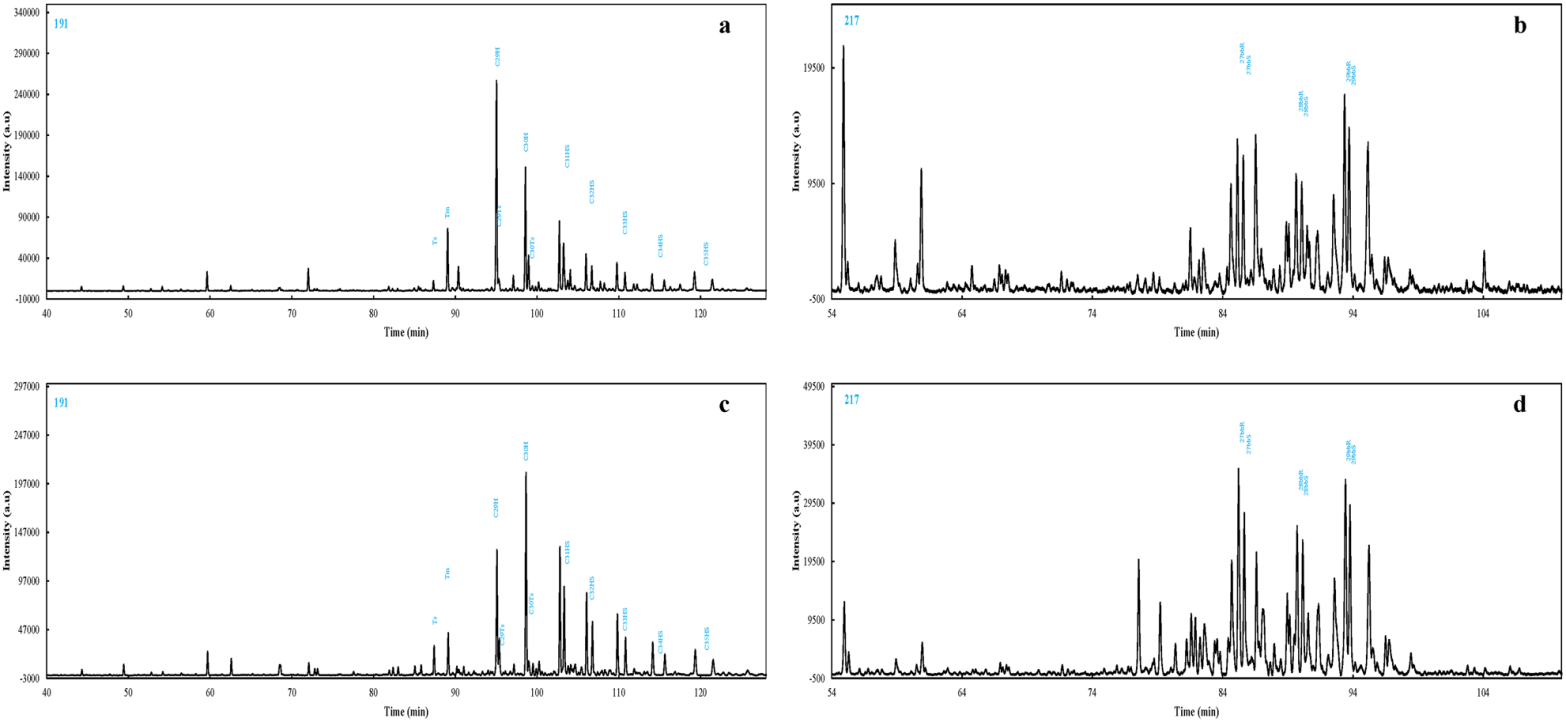
Mass chromatograms of m/z (**a**) 191 terpanes of hydrocarbon crude oil A, (**b**) 217 steranes of hydrocarbon crude oil A, (**c**) 191 terpanes of hydrocarbon crude oil B, (**d**) 217 steranes of hydrocarbon crude oil B.

To investigate the types of asphaltenes, asphaltene was extracted from two crude oil samples obtained from the Bangestan oil field in southern Iran, each with distinct specifications (refer to Table 1, Figs. 1, and 2). These extracted asphaltenes, designated as oils A and B, were subsequently used in the production of synthetic oils. Based on the analysis of the total acid number (TAN) and total base number (TBN) of the crude oil, asphaltene A was classified as acidic, whereas asphaltene B was classified as basic. For this study, five samples of synthetic oil, as indicated in Table 2, were utilized.

**Table 1:** Specifications of crude oils.

Specification		Result		Unit
		Crude Oil A	Crude Oil B	
Density@Ambient conditions		0.925	0.905	gr/cc
TBN		1.93	3.26	mgrKOH/gr
TAN		2.03	1.18	mgrKOH/gr
SARA	Saturates	43.4	50.01	% wt
	Aromatics	35.6	33.12	% wt
	Resins	12.9	10.47	% wt
	Asphaltenes	8.0	6.40	% wt
ICP-OES	Al	2.6	2.8	ppm
	Fe	0.3	0.2	ppm
	V	76	82	ppm
	Ni	20	22	ppm

**Table 2:** Characteristics of synthetic oil.

Symbol	Composition (by volume)	Asphaltene (wt.%)	Type of asphaltene
SYOil5%wtAsA	70% toluene 30% n-heptane	5	Crude Oil A (Acidic)
SYOil5%wtAsB	70% toluene 30% n-heptane	5	Crude Oil B (Basic)
SYOil1%wtAsA	70% toluene 30% n-heptane	1	Crude Oil A (Acidic)
SYOil1%wtAsB	70% toluene 30% n-heptane	1	Crude Oil B (Basic)
SYOil	70% toluene 30% n-heptane	0	–

To investigate the different components of oil in line with the objectives of this study, five samples of synthetic oil with varying compositions were prepared and evaluated, as outlined in Table 2. To ensure comparability among the different asphaltene samples, their percentages in the oil were determined to be equal. The determination of asphaltene percentages in the synthetic oils (1% and 5% by weight) was based on the following considerations:

1. To assess the effects of incorporating asphaltene into the oil, a sample of synthetic oil without asphaltene was included. Additionally, oils containing 1 wt.% of asphaltene (a percentage close to zero) were used to demonstrate the effect of asphaltene presence. Furthermore, two different asphaltene samples were employed to examine the influence of asphaltene type, specifically acidity and alkalinity.
2. To highlight differences in asphaltene percentages, the percentages were deliberately selected to be distinct from one another, enabling a clear differentiation.
3. The asphaltene percentages in the synthetic oils were intentionally reduced compared with those in the crude oils from which the asphaltenes were extracted. This choice allows for the evaluation of the study results on a realistic and field scale, aligning with the characteristics of the selected crude oils. Consequently, percentages below 6.4 wt.% (which is less than the asphaltene percentage of crude oil B, as indicated by the SARA analysis in Table 1) were considered.
4. The asphaltene percentages in the oil were determined to maintain the colloidal instability index (CII) (Eq. 1) within a stable range, with values below 0.7^70^. For 1 wt.% and 5 wt.% asphaltene oils, the calculated CII values were 0.34 and 0.39, respectively, indicating the stability of asphaltene in the oil.
  1 $$ CII = \frac{{{\text{Asphaltenes}} + {\text{Saturates}}}}{{{\text{Aromatics}} + {\text{Resins}}}} $$

### Bacteria characteristics

The bacteria used in this study were *G. Stearothermophilus* bio14 strains were isolated from reservoir fluid samples obtained from hydrocarbon reservoirs in southwestern Iran. The samples were collected from various well-sampling lines. The evaluation of the isolated bacterium has been conducted in previous studies, including those by Zargari et al.^69^, Sarafzadeh et al.^71,72^, and Abdi et al.^13^. These bacteria exhibit moderate halotolerance, ranging between 5 and 15%, indicating their moderate halophilic nature. *G. Stearothermophilus* (strain bio14) is well-known for its ability to produce biosurfactants^73^, which are resistant to elevated temperatures and salt levels^74^. These properties make the bacteria a compelling choice for this study. The injection of their biosurfactant offers significant advantages in carbonates and sandstones with moderate temperatures, including emulsification, reduction in IFT, alteration of wettability, and enhancement of pore-scale displacement^73^.

### Methodology

**Figure 3:**
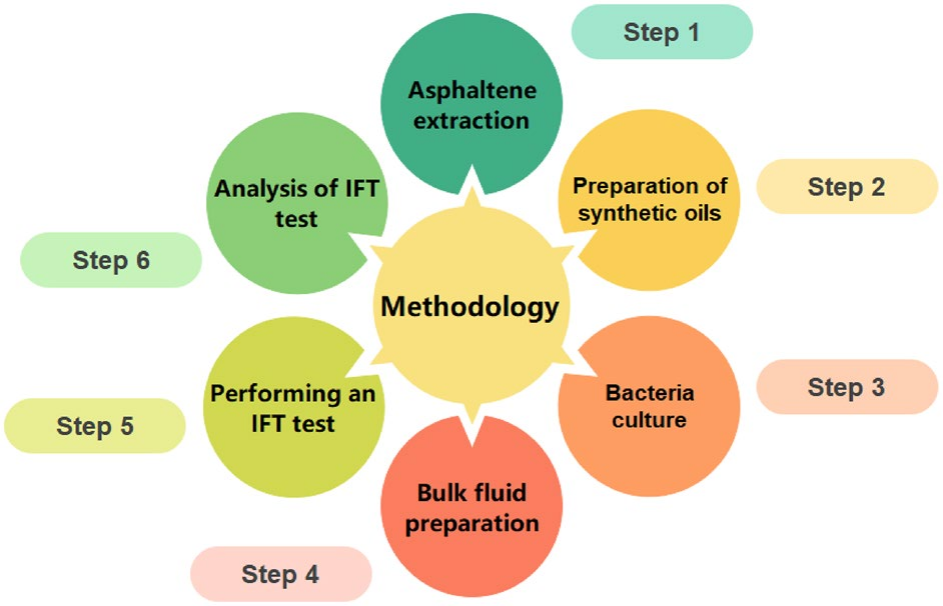
Summary of research methodology.

As shown in Fig. 3, the stages of the research can be summarized into six steps.

#### Preparation of synthetic oils

The first stage involved the extraction of asphaltene from crude oils using the IP-143 protocol method. Initially, a mixture of crude oil and heptane was prepared at a ratio of 1:40. The solution was stirred with a magnetic stirrer for 12 h to precipitate asphaltene. Following the 12-h stirring period, the solution was placed in the dark for an additional 12 h. This stirring and resting process was repeated three times. Subsequently, the crude oil and normal heptane solution were filtered using a vacuum filtration setup and Whatman 42 paper. The remaining solid particles on the Whatman paper were filtered and then transferred to the Soxhlet system for washing. The Soxhlet washing process consisted of two steps, starting with normal heptane, followed by toluene. The purpose of the normal heptane wash was to remove solid particles separated from the asphaltene. Note that both stages of Soxhlet washing were continued until the fluid within the Soxhlet extraction chamber became completely transparent. After the toluene was rinsed, the solution from the Soxhlet boiling flask was transferred to a glass container for toluene evaporation. The remaining solid particles after toluene evaporation constituted asphaltene, which would be used in subsequent stages.

In the second stage, a combination of n-heptane, toluene, and asphaltene was employed to prepare the synthetic oil samples based on Table 2. First, the desired percentage of asphaltene was added to toluene and thoroughly mixed for 30 min using a magnetic stirrer operating at a rotational speed ranging from 200 to 300 rpm. Subsequently, n-heptane was added to the resulting mixture, and the combined solution was stirred with a magnetic stirrer at a speed of 200–300 rpm for 15 min to ensure uniformity.

#### Cultivation of bacteria

Brain Heart Infusion (BHI) broth medium was used to cultivate single colonies of *G. Stearothermophilus* bio14 strains. The medium composition included 6 g/L BHI, 14.5 g/L gelatin, 3 g/L dextrose, 2.5 g/L Na_2_HPO_4_, 5 g/L KCl, and 6 g/L peptic digest of animal tissue. The medium was agitated at 150 rpm and 37 °C for 48 h until the OD600 nm value reached 2. Although the ideal condition for biosurfactant production is a medium like MSSO, *Bacillus* can still produce biosurfactants in the BHI medium, as reported previously^75^. Moreover, numerous studies have employed BHI as an effective medium for biosurfactant production by different bacteria types^76–79^. In previous investigations, BHI was proven to be effective in cultivating *G. Stearothermophilus* under harsh conditions^13,69,71,72^. According to Zargari et al.^69^, the isolation process was carried out. Initially, the cultured bacterial solution was evaluated for IFT. However, because of the turbidity of the bacterial solution, analyzing the IFT and capturing the image of the oil droplet within the bacterial bulk was not feasible. Therefore, the bacterial solution was mixed with deionized water at a one-to-one ratio to enable IFT evaluation. Deionized water was used to avoid the presence of polar components such as salt and to ensure reliable results. Additionally, in line with the study’s objective of combining bacteria with low-salinity water, an aqueous fluid compatible with low-salinity water was employed. Ultimately, the diluted bacterial solution reached an OD600 nm value of 0.8, which was used for IFT assessment. We conducted a limited-scale experiment using the previously mentioned biosurfactant to investigate its impact on reducing IFT and to understand the underlying mechanism.

#### Performing IFT tests

In the fourth step, the three salts (NaCl, MgCl_2_, and CaCl_2_) were prepared in two different environments: low-salinity water and a combination of low-salinity water with bacteria at salinity levels of 1000 and 5000 ppm. To prepare the aqueous solutions, salt was added to either deionized water or a bacterial solution. A magnetic stirrer operating at a maximum speed of 200–300 rpm was used to dissolve the salt and achieve a homogeneous solution within a time frame of up to 5 min.

**Figure 4:**
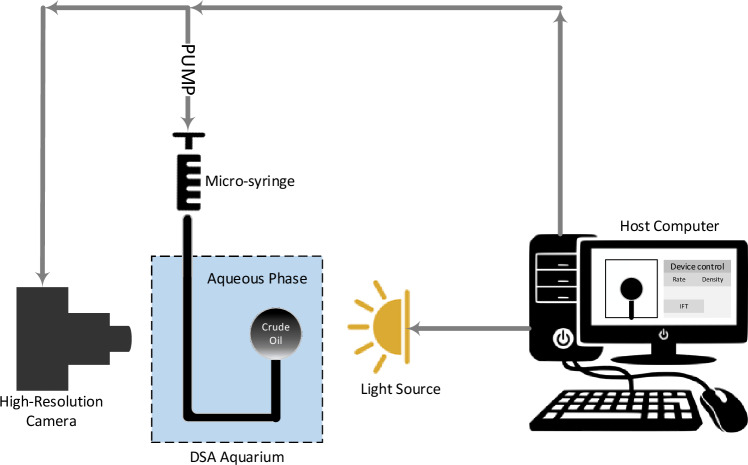
Schematic of the IFT device (DSA100-Krüss)^61^.

The IFT of the aqueous phase/oil was assessed using the Krüss DSA100 (Germany) device under ambient conditions, following the schematic shown in Fig. 4. This device comprises a drip injection pump, an aquarium base and holder, a glass aquarium, a light source, and a camera connected to a computer. The pendant drop method, as illustrated in Fig. 4, was employed to calculate the IFT. A microsyringe filled with oil, connected to an injection pump, was used to inject a specific volume of oil at a controlled flow rate determined by the computer settings. This injection process resulted in the formation of an oil drop at the tip of the needle within the aquarium, which was filled with aqueous phases. Using the device's light source, the camera captured an image of the oil drop formed at the needle’s tip. Subsequently, the drop picture was analyzed using the Fiji-win 64 ImageJ software (version 1.53e), and the IFT was computed using Eq. (2). The IFT between the two phases in Eq. (2). is governed by the equilibrium established among gravity, capillary forces, and the shape factor (H). The shape factor (H) is calculated using the parameter S = d_e_/d_s_ and the Young–Laplace equation, where d_e_ represents the equatorial droplet and d_s_ represents the diameter of the droplet at a distance de from the top of the droplet^80,81^. The densities of the assessed fluids employed for calculating the IFT were determined using the standard test method ASTM D1217-15 under ambient conditions. The gravitational acceleration (g) was also computed using the International Gravity Formula (IGF)^82,83^. The reported results in the current study were derived from the mean of two independent repetitions to demonstrate the reproducibility of the results.

2 $$ IFT = \frac{{\Delta \rho gd_{e}^{2} }}{H} $$

$\Delta \rho$: The disparity in density between the oil and the aqueous phase. g: Gravitational acceleration. d_e_: Equatorial diameter of the droplet. H: shape factor.

### Statistical analysis

The presented IFT results represent the mean value obtained from a minimum of two replicates. A summary of the statistical analysis conducted for all repetitions is provided in Table 3. Throughout the discussion section, the results are presented as the mean of multiple replicates, with error bars indicating the standard error (SE). Statistical analysis of all IFT data was performed using SigmaPlot software (version 14). Various statistical methods, including the Student–Newman–Keuls method, Tukey test, Dunn’s method, and Dunnett's method for analysis of variance (ANOVA) on ranks, were applied to analyze the significance level, and all tests yielded a significance level of P < 0.05.

**Table 3:** Statistical analysis of all IFT data obtained in this study.

Parameter	Size	Mean	Standard deviation (SD)	Standard error (SE)	Max	Min	Median
IFT (mN/m)	148	25.920	5.704	0.469	36.480	13.220	26.505

## Results and discussion

### Effect of bacteria on IFT reduction

**Figure 5:**
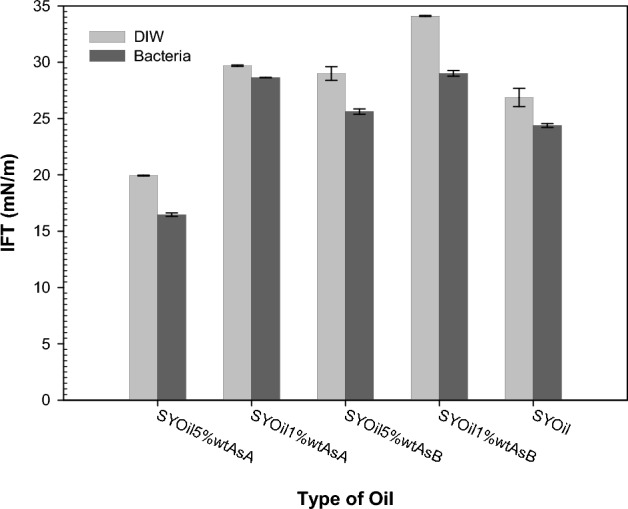
Effect of bacteria on the IFT reduction for different oils (different percentages and types of asphaltene).

Based on the results presented in Fig. 5 for five samples of synthetic oil with varying properties, the coexistence of bacteria and asphaltene with different percentages and structures demonstrates their potential to reduce IFT. The existence of bacteria results in the production of biosurfactants, which contribute to IFT reduction. Asphaltene can also function as a surfactant in the system. However, when bacteria and asphaltene coexist in the same system, the nature of the interaction between these two entities may vary depending on the asphaltene's structure and properties. Acidic asphaltene (A) and basic asphaltene (B) exhibit distinct characteristics.

The presence of bacteria leads to a greater reduction in IFT when basic asphaltene is present, as shown in Fig. 5 (SYOil1%wtAsB). This behavior is similar to the interaction described by Abdi et al.^61^, where the interaction between asphaltene and ion hydration shells was discussed. According to Abdi et al.^61^, the interaction between acidic asphaltene and anions, as well as between basic asphaltene and cations, primarily governs IFT behavior in oil/low-salinity water systems. Bacteria have a complex structure with polar regions that are both positively and negatively charged, resembling the interaction between asphaltene and various ions in brine. The further reduction in IFT observed for basic asphaltene in Fig. 5 suggests that the positively charged regions of bacteria have a dominant effect. This reduction is attributed to the interaction between the positively charged regions of bacteria and basic asphaltene, leading to the formation of intrinsic surfactants and increased production of biosurfactants at the oil-aqueous phase interface.

### Effect of salinity and salt type

**Figure 6:**
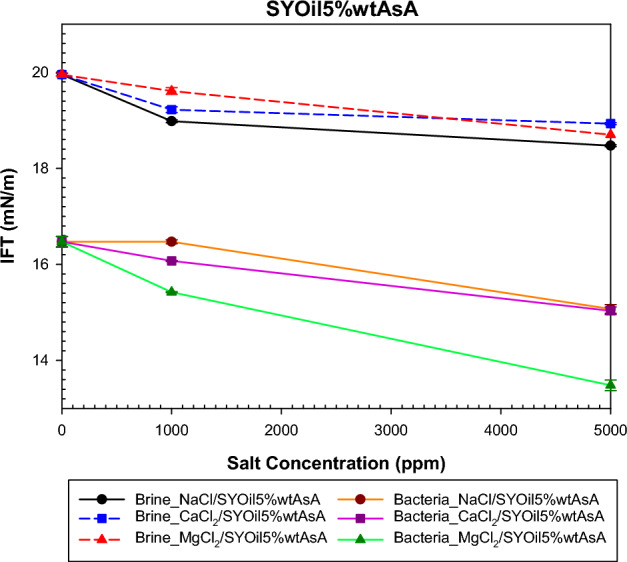
Effect of salinity and salt type on the IFT of low-salinity water and the combination of low-salinity water with biosurfactant for SYOil5%wtAsA.

**Figure 7:**
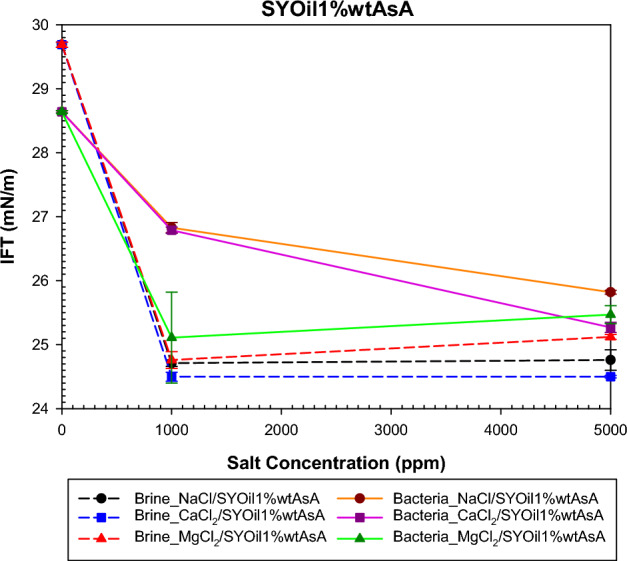
Effect of salinity and salt type on the IFT of low-salinity water and the combination of low-salinity water with biosurfactant for SYOil1%wtAsA.

**Figure 8:**
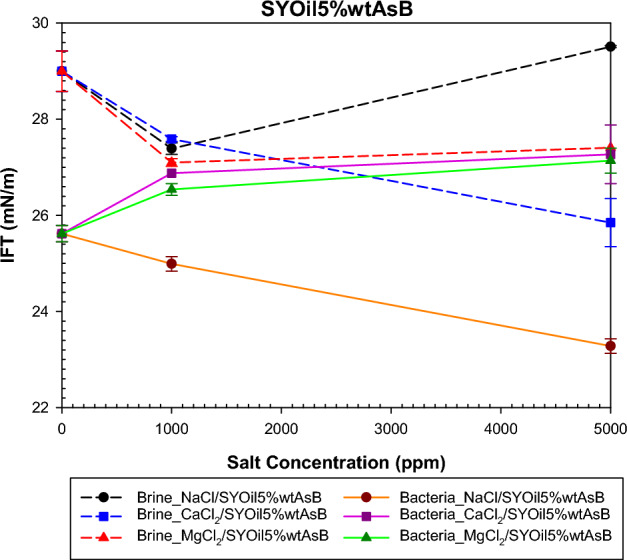
Effect of salinity and salt type on the IFT of low-salinity water and the combination of low-salinity water with biosurfactant for SYOil5%wtAsB.

**Figure 9:**
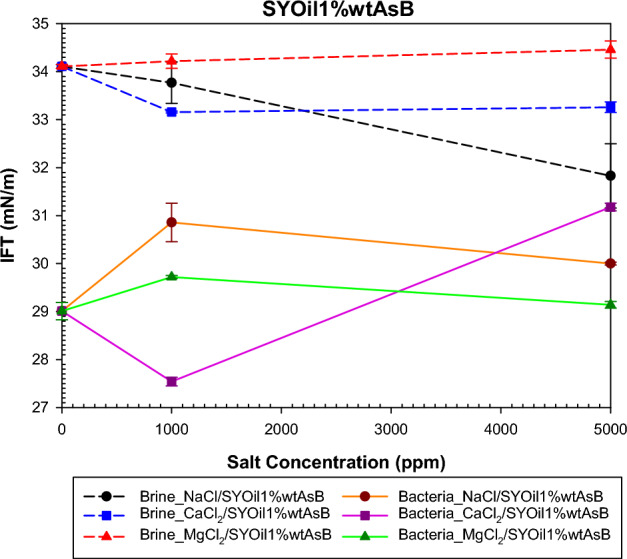
Effect of salinity and salt type on the IFT of low-salinity water and the combination of low-salinity water with biosurfactant for SYOil1%wtAsB.

The study examined the impact of salinity and salt type on IFT under both bacterial and non-bacterial conditions, with the oils specified in Table 2. The findings of this investigation are presented in Figs. 6, 7, 8 and 9. Abdi et al.^61^ proposed a mechanism explaining IFT in low-salinity water based on the interaction between the polar components of oil and ion hydration shells in water . Their findings indicate that IFT in oil/low -salinity water systems is influenced by factors such as ion hydration energy, ion concentration, and the type of polar components (acidic or basic) in the oil. This mechanism suggests that the interaction between oil and low-salinity water can result in different behaviors, as shown in Figs. 6, 7, 8 and 9.

Figure 6 demonstrates the ability of asphaltene in oil to reduce IFT. In the presence of low-salinity water, asphaltene acts as an intrinsic surfactant. When salts dissolve in water, the charged ions interact with the polar head of asphaltene, causing asphaltene to migrate toward the interface between the two phases. This behavior of asphaltene creates an intrinsic surfactant, which is attributed to the structure of the non-polar chains and polar components of the asphaltene. The presence of different ions in water can lead to varying interactions with asphaltene, affecting the results shown in Fig. 6 for SYOil5%wtAsA at 0, 1000, and 5000 ppm salinity of NaCl, CaCl_2_, and MgCl_2_ salts. The predominance of acidic components in SYOil5%wtAsA leads to stronger interactions with anions in low-salinity water. This interaction is evident in Fig. 6 at a salinity of 1000 ppm, where increased Cl^-^ ions result in reduced IFT. As the salinity of low-salinity water increases, with a simultaneous increase in both anions and cations, the repulsion and attraction forces also increase. This behavior causes simultaneous repulsion and attraction between the polar components of oil and the hydration shell of the ions, which differ at 5000 ppm salinity.

In the presence of bacteria within the system (Fig. 6), more complex interactions occur. The intricate structure of bacteria can result in diverse interactions with asphaltene. For SYOil5%wtAsA, the presence of bacteria demonstrated a greater reduction in IFT than low-salinity water. The observed discrepancy could be attributed to the generation of biosurfactants within the system by bacteria. IFT reduction is achieved through the combination of intrinsic surfactants formed by asphaltene and biosurfactants produced by bacteria. This favorable behavior of bacteria and asphaltene in reducing IFT relative to low-salinity water was observed across CaCl_2_, NaCl, and MgCl_2_ salts at salinities of 0, 1000, and 5000 ppm. The presence of polar components within bacteria combined with low salinity water facilitates interactions among bacteria, oil, low salinity water ions, and bacteria. As mentioned in the previous section, the positive charge within bacteria has a more dominant effect, enhancing their interaction with negatively charged. Acidic asphaltene also exhibits a stronger interaction with anions. This tendency for interaction is intensified by increasing the number of chloride ions. In Fig. 6, the number of chloride ions follows the sequence MgCl_2_ > CaCl_2_ > NaCl for NaCl, CaCl_2_, and MgCl_2_ salts, respectively. A higher number of Cl^-^ ions leads to an increased interaction with bacteria and asphaltene. Consequently, as the Cl^-^ ion count increases, more biosurfactants and intrinsic surfactants are produced, resulting in a lower IFT.

A decrease in the percentage of asphaltene A leads to different interactions with bacteria and ions in low-salinity water. The results of Fig. 7, compared with those of Fig. 6, indicate that reducing the percentage of acidic asphaltene, along with the presence of bacteria and salt at low salinity, results in an increase in IFT. When the bulk phase is low -salinity water, the results obtained for SYOil1%wtAsA demonstrate that the behavior of NaCl, MgCl_2_, and CaCl_2_ salts is similar. At low concentrations (1000 ppm), the behavior does not depend on the salt type. The low percentage of asphaltene A reduces its likelihood of being present at the interface. For 1000 ppm, the results show the lowest IFT, which is attributed to the increased interaction of asphaltene A with system components, leading to the formation of intrinsic surfactants. Increasing the salinity from 1000 to 5000 ppm revealed that the low percentage of asphaltene in the oil diminishes the potential for intrinsic surfactant formation and has minimal impact on the changes in IFT above 1000 ppm (Fig. 7). In the absence of salt, bacteria lead to a reduction in IFT for SYOil1%wtAsA, which is attributed to the formation of biosurfactants. However, the behavior of the system becomes more complex when salt is added. The IFT behavior, as depicted in Fig. 7, is similar to that of SYOil5%wtAsA in the presence of bacteria and a salinity of 1000 ppm salts, depending on the number of Cl ions. However, at a salinity of 5000 ppm, the repulsion between the cation and asphaltene A causes a different IFT behavior compared with that observed in SYOil5%wtAsA.

Figure 8 illustrates the effect of salinity and salt type on SYOil5%wtAsB. Analysis of the TAN and TBN values presented in Table 1 reveals that the predominant polar compounds of oil B are basic. Most asphaltene in crude oil B can be considered basic. According to the mechanism proposed by Abdi et al.^61^, the interaction between B oils (SYOil5%wtAsB and SYOil1%wtAsB) and cations can have a more significant impact on IFT behavior. The IFT behavior, as shown in Fig. 8, depends on the hydration energy of the cations and the concentration of the anions. Cations promote asphaltene adsorption at the interface, whereas anions cause repulsion. The balance between attraction and repulsion is influenced by the hydration energy and the quantity of ions. At 1000 ppm salinity, the presence of ions enhances their interaction with asphaltene, resulting in reduced IFT compared with that at 0 salinity. As salinity increases from 1000 to 5000 ppm, the interactions become more complex. The order of cation hydration energy is Mg^2+^ > Ca^2+^ > Na^+^, while the order of chlorine ion concentration is MgCl_2_ > CaCl_2_ > NaCl. Higher cation hydration energy leads to greater asphaltene adsorption, whereas a higher concentration of anions causes increased asphaltene repulsion at the interface. Consequently, NaCl exhibits the least interaction with asphaltene at 5000 ppm because of its lower cation hydration energy, hindering the formation of intrinsic surfactants. Despite Mg^2+^ having a higher hydration energy than Ca^2+^, the higher concentration of Cl^-^ ions in MgCl_2_ results in an elevated IFT, surpassing that of CaCl_2_.

The presence of bacteria in the aqueous phase and their affinity for basic asphaltene result in the formation of biosurfactants, which reduce IFT in the absence of salt. Bacteria interact with cations and anions, leading to their absorption and repulsion at the interface between phases. The presence of cations with higher hydration energy in the bulk of the bacteria enhances their interaction. Therefore, as shown in Fig. 8, when Mg^2+^ and Ca^2+^ cations are present, the interaction between the biosurfactant and the hydration shell of these cations leads to an increase in IFT. However, Na^+^ cations, due to their lower hydration energy, this repulsion is not sufficient to overcome the interaction between the anion, bacteria, and basic asphaltene, resulting in the formation of intrinsic surfactants and biosurfactants. Changes in IFT for salts in the presence of bacteria are influenced by the number of ions and the interactions between ions, bacteria, and asphaltene, as well as the interaction between asphaltene and bacteria.

The findings depicted in Fig. 9, regarding the bulk of low salinity water, demonstrate the correlation between the outcomes and the number of Cl^−^ ions. The interaction between basic asphaltene and cations/anions results in the adsorption and desorption of asphaltene at the interface of the two phases, respectively. The impact of anions on the interaction with asphaltene intensifies with increasing salt concentrations. The trend of changes in IFT with increasing salt salinity shows an increasing trend for MgCl_2_, a decreasing trend for NaCl, and a dual behavior (decreasing-increasing) for CaCl_2_. The sequence of Cl^-^ ions is MgCl_2_ > CaCl_2_ > NaCl, which influences their interaction with asphaltene and the behavior of IFT. A higher number of anions leads to greater removal of asphaltene from the interface. Therefore, the increase in IFT with increasing salinity for MgCl_2_ can be attributed to the higher number of Cl^-^ ions compared to CaCl_2_ and NaCl. While NaCl has the lowest chlorine ion content among the three salts, which leads to less removal of the inherent surfactant in the two-phase interface compared with CaCl_2_ and MgCl_2_. In the case of CaCl_2_, its Cl^-^ ion content is higher than that of NaCl but lower than that of MgCl_2_, leading to a dual behavior in response to increasing salinity.

The interaction in their presence of bacteria is also influenced by the number of ions and their hydration energy. The order of cations is Na^+^ > Mg^2+^ > Ca^2+^, indicating that in the presence of a lower number of cations, a greater amount of biosurfactant can be formed at a salinity of 1000 ppm. As the salinity increases, it leads to a variety of interactions because it increases the number of Cl^-^ ions compared to cations, resulting in the behavior of IFT not exhibiting a specific trend.

### Effect of asphaltene percentage

**Figure 10:**
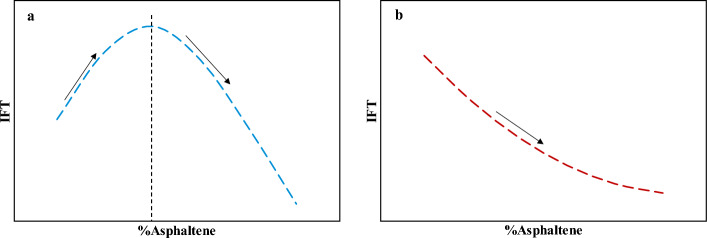
Schematic of changes in IFT with increasing percentage of asphaltenes.

To investigate asphaltene's effect on the IFT of low -salinity water and bacteria, weight percentages of 0, 1, and 5 of asphaltene were examined (Tables 4 and 5). The interaction between the ion hydration shell in low-salinity water and asphaltene leads to an IFT reduction as the concentration of asphaltene in oil increases. This behavior was observed for both acidic and basic asphaltene samples (Fig. 10-b). The results presented in Table 4 demonstrate that, under identical conditions of salt type, salinity, and asphaltene percentage, the IFT consistently shows lower values for acidic asphaltene than for basic asphaltene. This behavior can be attributed to the higher number of Cl^-^ anions, which exceed the number of cations in CaCl_2_, NaCl, and MgCl_2_ salts. In addition, acidic asphaltene has a greater tendency to interact with the hydration shell of anions. Therefore, according to Table 4, acidic asphaltene consistently displays a lower IFT than basic asphaltene under the same conditions.

**Table 4:** Changes in IFT for different percentages and types of asphaltene in the absence of bacteria.

Bulk	Salt	Salinity	IFT $\pm$ SE [mN/m]					
			wt.% Asphaltene A			wt.% Asphaltene B		
			0	1	5	0	1	5
DIW	–	–	26.87 $\pm$ 0.40	29.69 $\pm$ 0.04	19.95 $\pm$ 0.03	26.87 $\pm$ 0.40	34.11 $\pm$ 0.04	29.00 $\pm$ 0.42
Brine	NaCl	1000 ppm	35.42 $\pm$ 0.75	24.71 $\pm$ 0.05	18.98 $\pm$ 0.03	35.42 $\pm$ 0.75	33.77 $\pm$ 0.43	27.39 $\pm$ 0.12
		5000 ppm	33.64 $\pm$ 0.98	24.76 $\pm$ 0.16	18.47 $\pm$ 0.02	33.64 $\pm$ 0.98	31.83 $\pm$ 0.67	29.51 $\pm$ 0.02
	CaCl_2_	1000 ppm	34.32 $\pm$ 0.24	24.50 $\pm$ 0.07	19.22 $\pm$ 0.06	34.32 $\pm$ 0.24	33.16 $\pm$ 0.02	27.59 $\pm$ 0.05
		5000 ppm	33.67 $\pm$ 0.02	24.50 $\pm$ 0.02	18.93 $\pm$ 0.02	33.67 $\pm$ 0.02	33.26 $\pm$ 0.11	25.85 $\pm$ 0.50
	MgCl_2_	1000 ppm	35.12 $\pm$ 0.18	24.76 $\pm$ 0.13	19.61 $\pm$ 0.07	35.12 $\pm$ 0.18	34.22 $\pm$ 0.15	27.10 $\pm$ 0.08
		5000 ppm	35.50 $\pm$ 0.19	25.12 $\pm$ 0.04	18.70 $\pm$ 0.00	35.50 $\pm$ 0.19	34.46 $\pm$ 0.18	27.41 $\pm$ 0.00

**Table 5:** Changes in IFT for different percentages and types of asphaltene in the presence of bacteria.

Bulk	Salt	Salinity	IFT $\pm$ SE [mN/m]					
			wt.% Asphaltene A			wt.% Asphaltene B		
			0	1	5	0	1	5
Bacteria	–	–	24.39 $\pm$ 0.12	28.64 $\pm$ 0.02	16.47 $\pm$ 0.11	24.39 $\pm$ 0.12	29.01 $\pm$ 0.18	25.62 $\pm$ 0.17
Bacteria + Salt	NaCl	1000 ppm	29.13 $\pm$ 0.21	26.83 $\pm$ 0.08	16.47 $\pm$ 0.04	29.13 $\pm$ 0.21	30.86 $\pm$ 0.40	24.99 $\pm$ 0.15
		5000 ppm	27.12 $\pm$ 0.73	25.82 $\pm$ 0.03	15.07 $\pm$ 0.09	27.12 $\pm$ 0.73	30.00 $\pm$ 0.03	23.28 $\pm$ 0.15
	CaCl_2_	1000 ppm	24.06 $\pm$ 0.78	26.79 $\pm$ 0.05	16.07 $\pm$ 0.01	24.06 $\pm$ 0.78	27.54 $\pm$ 0.09	26.88 $\pm$ 0.00
		5000 ppm	27.24 $\pm$ 0.20	25.27 $\pm$ 0.08	15.03 $\pm$ 0.08	27.24 $\pm$ 0.20	31.18 $\pm$ 0.08	27.27 $\pm$ 0.61
	MgCl_2_	1000 ppm	25.65 $\pm$ 0.09	25.11 $\pm$ 0.71	15.42 $\pm$ 0.01	25.65 $\pm$ 0.09	29.72 $\pm$ 0.03	26.54 $\pm$ 0.12
		5000 ppm	25.83 $\pm$ 0.99	25.47 $\pm$ 0.14	13.48 $\pm$ 0.11	25.83 $\pm$ 0.99	29.14 $\pm$ 0.07	27.14 $\pm$ 0.26

The results presented in Fig. 5 and the aforementioned findings indicate that bacteria exhibit a higher interaction tendency with anions and negative charges. In addition, asphaltene A, which is primarily acidic, exhibits a stronger interaction with the hydration shell of the anions. Consequently, the elevated concentration of Cl^-^ ions in each of the CaCl_2_, NaCl, and MgCl_2_ salts, compared with the number of cations, increases the likelihood of their interaction with asphaltene A and bacteria. This inclination of anion interaction with bacteria and asphaltene A leads to the formation of biosurfactants and intrinsic surfactants, which is primarily facilitated by the higher number of Cl^-^ ions in the case of acidic asphaltene. According to Table 5, the increase in the percentage of asphaltene A suggests that the likelihood of interaction with anions will likely increase, promoting the formation of intrinsic surfactants and resulting in a reduction in IFT with the increase in the percentage of asphaltene A (except one case). A comparison of the results between low-salinity water and bacterial systems for NaCl, CaCl_2_, and MgCl_2_ reveals that a lower percentage of asphaltene corresponds to a lower IFT in low-salinity water. The observed phenomenon can be attributed to the existence of bacteria, the formation of biosurfactants initially dominates, leading to a decrease in IFT (in the absence of asphaltene). However, upon the addition of up to 1% asphaltene to the oil, the interaction between salt, asphaltene, and bacteria, as well as the interaction between asphaltene and bacteria, results in a diminished inclination to generate intrinsic surfactants. In the presence of bacteria and a lower percentage of asphaltene A, this decrease in intrinsic surfactant formation leads to higher IFT values compared with low-salinity water (Fig. 10-a). Conversely, an increase in the percentage of asphaltene A in the system decreases IFT in the bacterial + salt bulk system compared with low-salinity water. This decrease in IFT is attributed to the increased tendency to form more intrinsic surfactants at the two-phase interface due to the presence of a higher amount of asphaltene.

Asphaltene B exhibits a distinct behavior compared with asphaltene A in its interaction with ions and bacteria. The interaction of the hydration shell of cations and anions with basic asphaltene B leads to the absorption and removal of asphaltene at the interface, respectively. However, the bacteria *G. stearothermophilus* has an opposite interaction with anions and cations compared to asphaltene B. Furthermore, the interaction between asphaltene B (basic) and the bacteria is more pronounced. This contrasting pattern of interactions results in a lower formation of intrinsic surfactants and biosurfactants at the two-phase interface when the percentage of asphaltene B is low, such as 1 wt.% (Fig. 10-a). In this case, the interaction between Cl^−^ ions, which are more abundant than cations, and asphaltene B, as well as the interaction between cations and bacteria, hinders the formation of intrinsic surfactants and biosurfactants. However, as the percentage of asphaltene B increases beyond 1 wt.%, the likelihood of interaction between asphaltene and the bacteria itself increases, leading to an increased formation of intrinsic surfactants and biosurfactants at the interface. As indicated in Tables 4 and 5, the IFT values for various salts and salinities can be influenced by factors such as ion type, salinity, and asphaltene percentage. These observations apply to both low-salinity water and the combination of bacteria and low-salinity water. The findings demonstrate that the interaction between asphaltene and salt is contingent on the specific percentage of asphaltene, the type of salt, and the salinity level.

### Effect of asphaltene type

As shown in Tables 4 and 5, both acidic and basic asphaltene exhibit similar behaviors in low-salinity water. Bacterial activity can be described as the production of biosurfactants that reduce IFT in the absence of asphaltene. However, in the presence of basic asphaltene, the biosurfactant interacts inversely with the intrinsic surfactant generated by the asphaltene. When comparing the IFT values, it is observed that an increasing percentage of basic asphaltene results in a smaller reduction in IFT for the bulk of bacteria compared with low-salinity water.

The nature of the polar components found in oil, including their acidity and alkalinity, significantly influences their interaction with the aqueous phase. Asphaltene A exhibited a tendency to interact with negative charges because of the prevalence of its acidic components, whereas asphaltene B tended to interact with positive charges because of the predominance of basic components. The simultaneous presence of bacteria and salt in the aqueous phase alters the interaction between asphaltene and ions in the aqueous phase. As discussed earlier, there is a stronger interaction between bacteria and basic asphaltene and a higher tendency for bacteria to interact with negative charges. Consequently, anions in the aqueous phase, with their higher abundance compared with cations in CaCl_2_, NaCl, and MgCl_2_ salts, result in a greater interaction with acidic asphaltene and bacteria. The simultaneous interaction of anions with asphaltene A and bacteria leads to the formation of intrinsic surfactants and biosurfactants, which explains the decreasing trend in IFT observed for asphaltene A as the percentage of asphaltene increases. The similarity in the behavior of IFT with an increasing percentage of asphaltene in low -salinity water and bacterial systems, as indicated in Tables 4 and 5, suggests that the interaction between anions, acidic asphaltene, and bacteria follows a similar trend.

The interaction between ions in the aqueous phase and basic asphaltene differs from that of acidic asphaltene. Cations in the aqueous bulk tend to attract asphaltene B to the two-phase interface, whereas anions repel it from the interface. Conversely, the interaction of bacteria with anions and cations is contrary to their interaction with asphaltene B. However, the interaction at the two-phase interface between bacteria and asphaltene B promotes the absorption of both biosurfactants and intrinsic surfactants. The contrasting interactions between anions and cations with bacteria and asphaltene B, as well as the interaction between asphaltene B and bacteria, result in an IFT behavior that does not exhibit a clear trend concerning increasing the percentage of asphaltene B.

The evaluation of many factors, including salinity, type of salt, TAN and TBN of the oil, percentage of asphaltene, and the presence or absence of bacteria (as shown in Tables 4 and 5), revealed that all these parameters have an impact on IFT. Consequently, a multiple linear regression model was developed to analyze the influence of these parameters on IFT. Equation (3) represents the multiple linear models, and a summary of the statistical analysis for the independent parameters is provided in Table 6. The regression model successfully passed both the normality test, conducted using the Shapiro–Wilk method, and the constant variance test, performed using the Spearman Rank Correlation method. These tests confirmed that the data is normally distributed around the regression line, indicating the suitability of the regression model for data evaluation. Furthermore, the P and F values from the ANOVA of the multiple linear regression model for IFT, as presented in Table 7, further support the appropriateness of the regression model. Additionally, the statistical evaluation of the R, R^2^, and R^2^_adj_ parameters of the model yielded values of 0.922, 0.850, and 0.833, respectively. These values indicate that the regression model provides a good description of the relationship between the independent and dependent variables. Moreover, a summary of the statistical analysis for the independent parameters of the regression model, as outlined in Table 6, highlights the significant influence of the interaction between bacteria and asphaltene, as well as the type of polar components of asphaltene, on IFT.

3 $$ \begin{aligned} {\varvec{IFT}} & = 31.586 - \left( {3.949 \times OD600_{nm} } \right) - \left( {4.41 \times 10^{ - 5} \times MgCl_{2} } \right) \\ & \;\;\; - \left( {5.71 \times 10^{ - 5} \times CaCl_{2} } \right) - \left( {7.66 \times 10^{ - 5} \times NaCl} \right) \\ & \;\;\; - \left( {1.668 \times Asphaltene} \right) - \left( {4.405 \times TAN} \right) + \left( {2.931 \times TBN} \right) \\ \end{aligned} $$

IFT: Interfacial tension (mN/m). OD600_nm_: Bacteria concentration. MgCl_2_, CaCl_2_, and NaCl: Salt concentration in the aqueous phase (ppm). Asphaltene: Asphaltene percentage in oil (wt.%). TAN and TBN: Total acid number and total base number (mgrKOH/gr).

**Table 6:** Summary of the statistical analysis of independent parameters for multiple linear regression of IFT.

	Coefficient	SE	t	P	Variance Inflation Factor (VIF)
Constant	31.586	0.746	42.322	< 0.001	–
OD600_nm_	− 3.949	0.683	− 5.780	< 0.001	1.000
MgCl_2_	− 0.0000441	0.000173	− 0.255	0.799	1.192
CaCl_2_	− 0.0000571	0.000173	− 0.330	0.742	1.192
NaCl	− 0.0000766	0.000173	− 0.443	0.659	1.192
Asphaltene	− 1.668	0.153	− 10.918	< 0.001	1.450
TAN	− 4.405	0.444	− 9.925	< 0.001	1.469
TBN	2.931	0.277	10.562	< 0.001	1.476

**Table 7:** ANOVA of the multiple linear regression model for IFT.

	Degrees of Freedom (DF)	The sum of Squares (SS)	Mean Square (MS)	F	P
Regression	7	1840.178	262.883	50.294	< 0.001
Residual	62	324.072	5.227	–	–
Total	69	2164.250	31.366	–	–

## Summary and conclusions

The research demonstrates that G. stearothermophilus can reduce IFT even in the absence of salt. This capacity is demonstrated in the presence and absence of both acidic and basic asphaltene. Biosurfactant formation at the aqueous phase/oil interface is a key factor in reducing IFT due to *G. stearothermophilus*. Nevertheless, the significant reduction in IFT was unexpected because of the low concentration of the biosurfactant. Higher percentages of acidic and basic asphaltenes lead to increased interaction between bacteria and asphaltene, resulting in further reduction of IFT. The interaction of acidic asphaltene with NaCl, MgCl_2_, and CaCl_2_ salts in the presence of *G. stearothermophilus* is stronger, leading to lower IFT compared to basic asphaltene. This behavior is attributed to the tendency of acidic asphaltene and bacteria to react with Cl anions. The presence of a higher number of Cl^−^ ions enhances their interaction with acidic asphaltene and bacteria, resulting in the production of more intrinsic surfactants and biosurfactants.

In the presence of *G. stearothermophilus* and various salts, the interaction between asphaltene, bacteria, and salt depends on the type and percentage of asphaltene. Increasing the percentage of acidic asphaltene (A) in the presence of CaCl_2_, NaCl, and MgCl_2_ salts results in behavior similar to low-salinity water, with a decreasing trend in IFT. In low-salinity water, a lower IFT is observed compared with the bulk containing *G. stearothermophilus* and 1 wt.% acidic asphaltene. In the absence of asphaltene, biosurfactant formation dominates IFT behavior when the bulk contains *G. stearothermophilus*. The interaction of acidic asphaltene with salt and *G. stearothermophilus* bacteria with the addition of acidic asphaltene up to 1 wt.% to oil, has reduced the tendency to form intrinsic surfactants compared to low-salinity water. Reducing the intrinsic surfactant in the bulk of bacteria compared with low-salinity water will increase the IFT. Increasing the percentage of acidic asphaltene increases the formation of intrinsic surfactants, which becomes the dominant factor determining the system behavior. The presence of more intrinsic surfactant, along with biosurfactant, leads with lower IFT in bulk-containing bacteria compared with low-salinity water for oil with 5 wt.% acidic asphaltene.

For basic asphaltene (B), the presence of bacteria always reduces IFT compared with low-salinity water. Basic asphaltene and bacteria consistently promote the formation of more biosurfactants and intrinsic surfactants at the aqueous phase/oil interface. With all salts (NaCl, MgCl_2_, and CaCl_2_), increasing the percentage of basic asphaltene increases the formation of intrinsic surfactants and biosurfactants.

## Data Availability

All data generated or analyzed during this study have been included in this published article.
