# Author Correction: Investigating the mechanism of interfacial tension reduction through the combination of low-salinity water and bacteria

**DOI:** 10.1038/s41598-024-64861-4

**Published:** 2024-06-18

**Authors:** Arastoo Abdi, Behnam Ranjbar, Yousef Kazemzadeh, Farzaneh Aram, Masoud Riazi

**Affiliations:** 1 IOR/EOR Research Institute, Enhanced Oil Recovery (EOR) Research Center, Shiraz University, Shiraz, Iran; 2 Department of Petroleum Engineering, Faculty of Petroleum, Gas, and Petrochemical Engineering, Persian Gulf University, Bushehr, Iran; 3 Biotechnology Institute, College of Agriculture, Shiraz University, Shiraz, Iran; 4 School of Mining and Geosciences, Nazarbayev University, Kabanbay Batyr 53, Astana, 010000 Kazakhstan

Correction to: *Scientific Reports* 10.1038/s41598-024-62255-0, published online 18 May 2024

In the original version of this Article Arastoo Abdi and Behnam Ranjbar were omitted as equally contributing authors. In addition, Masoud Riazi was omitted as a corresponding author. Correspondence and requests for materials should also be addressed to mriazi180@gmail.com.

The original Article has been corrected.

